# The venous ulcer continues to be a clinical challenge: an update

**DOI:** 10.1186/s41038-018-0119-y

**Published:** 2018-06-15

**Authors:** Ting Xie, Junna Ye, Kittipan Rerkasem, Rajgopal Mani

**Affiliations:** 1 Wound Healing Centre at Emergency Department, Shanghai Ninth People’s Hospital, Shanghai Jiao Tong University School of Medicine, Shanghai, China; 2 Department of Rheumatology and Immunology, Ruijin Hospital, Shanghai Jiao Tong University School of Medicine, Shanghai, China; 3 NCD Centre of Excellence, Research Institute of Health Sciences, Chiang Mai University, Chiang Mai, Thailand; 4 NCD Centre and Department of Surgery, Faculty of Medicine, Chiang Mai University, Chiang Mai, Thailand; 5 Academic Division of Human Health and Development, Faculty of Medicine, University of Southampton, Southampton, UK; 6 Shanghai Jiao Tong University, Shanghai Jiao Tong School of Medicine, Shanghai, China

**Keywords:** Venous ulcer, Venous hypertension, Lipodermatosclerosis, Microcirculatory dysfunction, Technology guidelines

## Abstract

Venous ulcers are a common chronic problem in many countries especially in Northern Europe and USA. The overall prevalence of this condition is 1% rising to 3% in the over 65 years of age. Over the last 25 years, there have been many developments applicable to its diagnosis and treatment. These advances, notwithstanding healing response and recurrence, are variable, and the venous ulcer continues to be a clinical challenge.

The pathogenesis of venous ulcers is unrelieved or ambulatory venous hypertension resulting mostly from deep venous thrombosis leading to venous incompetence, lipodermatosclerosis, leucocyte plugging of the capillaries, tissue hypoxia and microvascular dysfunction. It is not known what initiates venous ulcers. Triggers vary from trauma of the lower extremity to scratching to relieve itchy skin over the ankle region. Venous ulcers can be painful, and this condition presents an increasing burden of care. A systematic analysis of the role of technology used for diagnosis and management strongly supports the use of compression as a mainstay of standardised care. It further shows good evidence for the potential of some treatment procedures to accelerate healing. This article reviews the pathogenetic mechanisms, current diagnostic methods and standard care and its limitations.

## Background

**Fig. 1:**
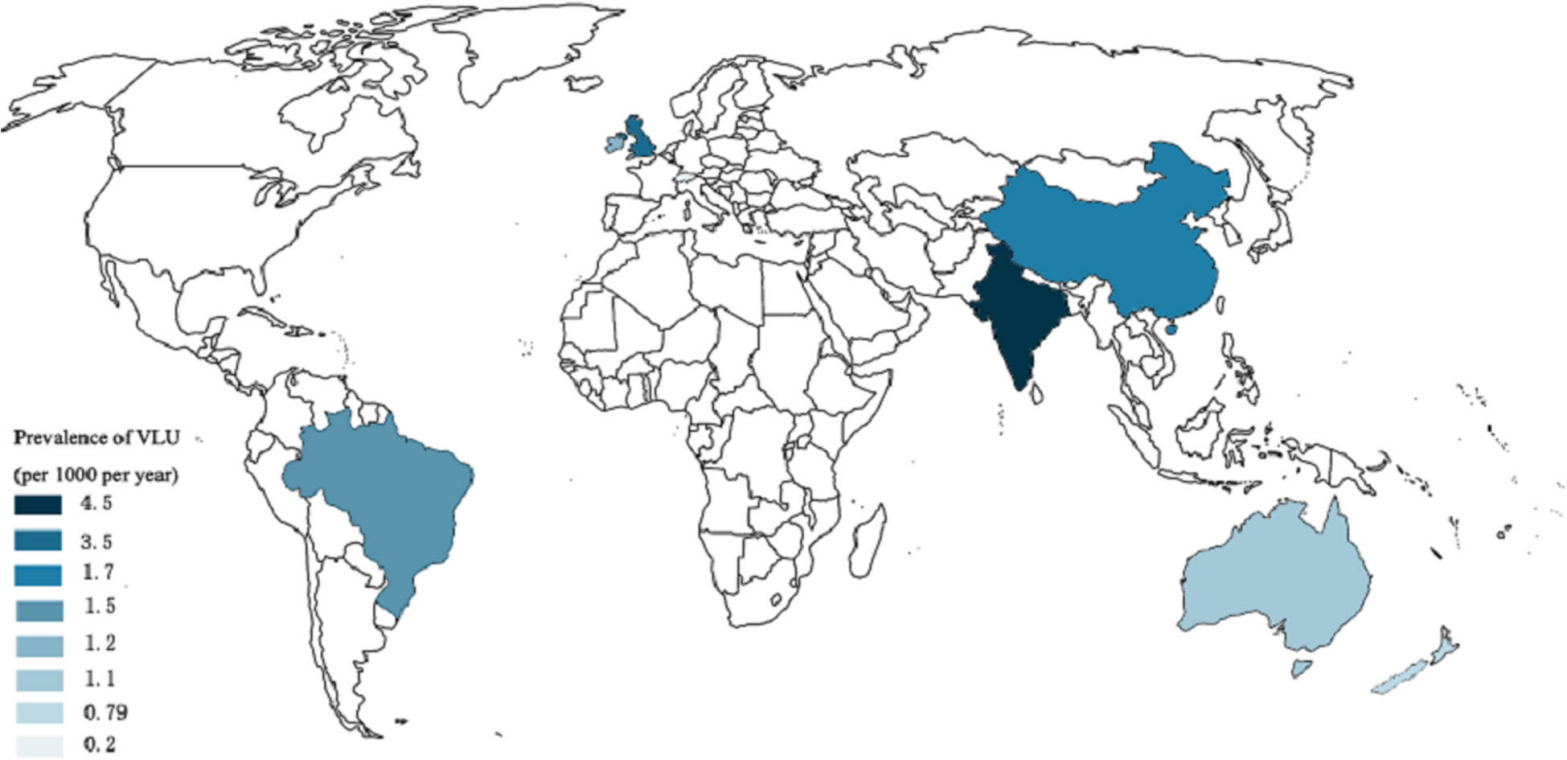
Prevalence of venous leg ulcers (VLU_s_) in different countries across the world. A darker colour is used to represent higher prevalence. (Prevalence was reported per 1000 individuals per year)

Venous leg ulcers (VLU_s_) are a major clinical challenge. VLU_s_ are among the most common chronic wounds presenting on the lower extremities and feet in man with prevalence up to 3% in the over 65 years of age in the UK [1]. Worldwide reported prevalence data are graphically presented in Fig. 1 [2–8]. Recently, Ting Xie and colleagues reported that the VLU_s_ are the greatest proportion of chronic wounds in the population over 60 years of age from a retrospective analysis of 5 years’ data on chronic wounds managed in Shanghai, China [9]. This finding could be significant since society is facing an increasing burden of cost of managing VLU_s_. Guest et al., after conducting a case control study of 1000 patients with chronic wounds (and 1000 age-matched controls without wounds), reported that the cost of managing all chronic wounds and associated morbidities was £5.3 billion to the UK exchequer [10].

**Fig. 2:**
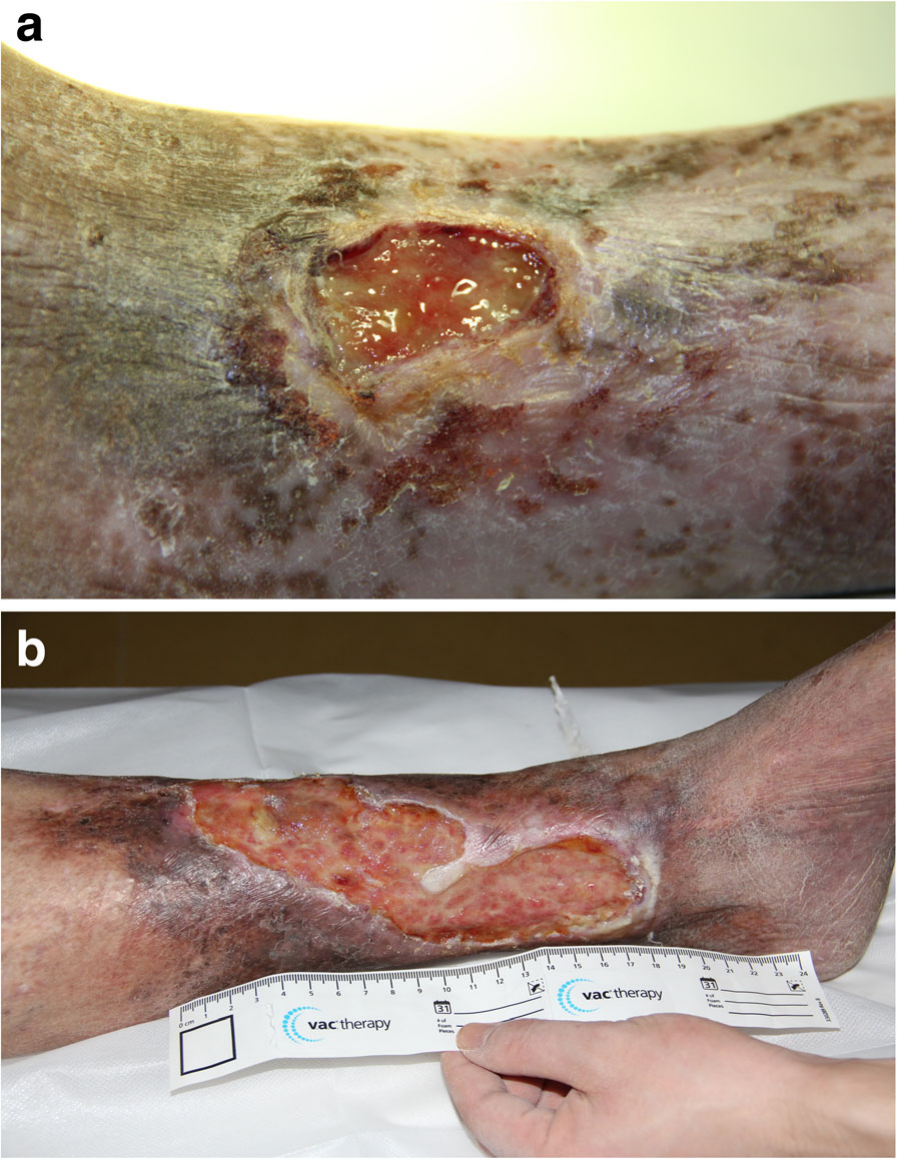
**a** Typical appearance of patient with lipodermatosclerosis. The skin is flaky and there is a brownish discoloration. The skin can have a waxy feel to it. **b** A venous ulcer on the medial aspect of the leg

**Fig. 3:**
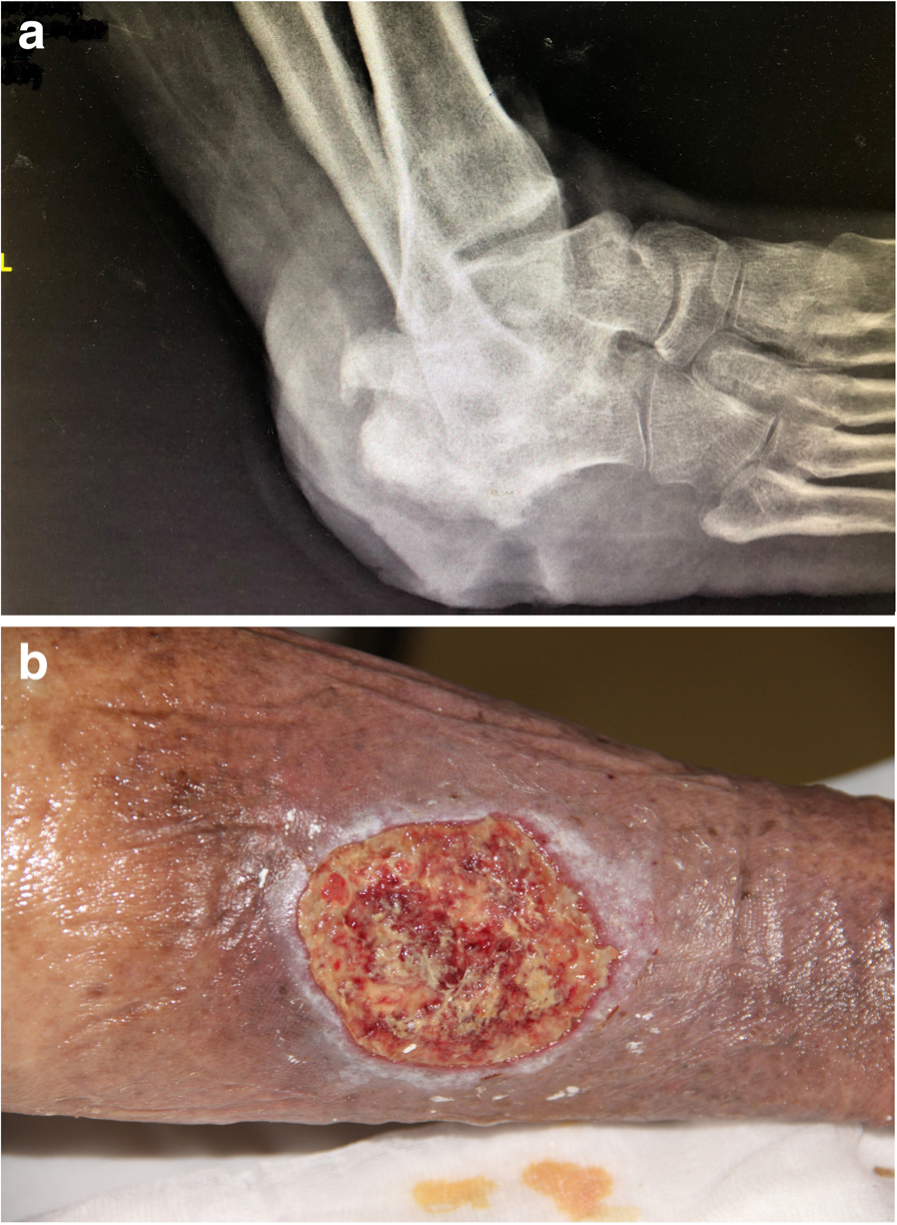
**a** A plain X-ray film of a patient with a long-standing venous leg ulcer (VLU). Notice the extensive loss of bone due to infection (osteomyelitis). On account of recurrent episodes of sepsis, the patient received a leg amputation. **b** A long-standing VLU almost across the lower calf region. Notice the raised edges of the ulcer: a biopsy to exclude cancer proved to be a squamous cell carcinoma

VLU_s_ present in the skin over the ankles, either on the inner or outer aspect of the malleolus, can be painful [11] and are often colonised, with underlying comorbidities such as rheumatoid arthritis and diabetes as shown in Figs. 2 and 3. The treatment of VLU_s_ is based on standardised care which relies on a reliable diagnosis, compression and local wound care. The healing of VLU_s_ is variable, recurrence and common [12]. The aim of this paper is to review the pathogenesis and evidence-based options for *standardised care*. Standardised care is based on getting a good diagnosis and treatment of the underlying cause.

## Review

### Pathogenesis

**Fig. 4:**
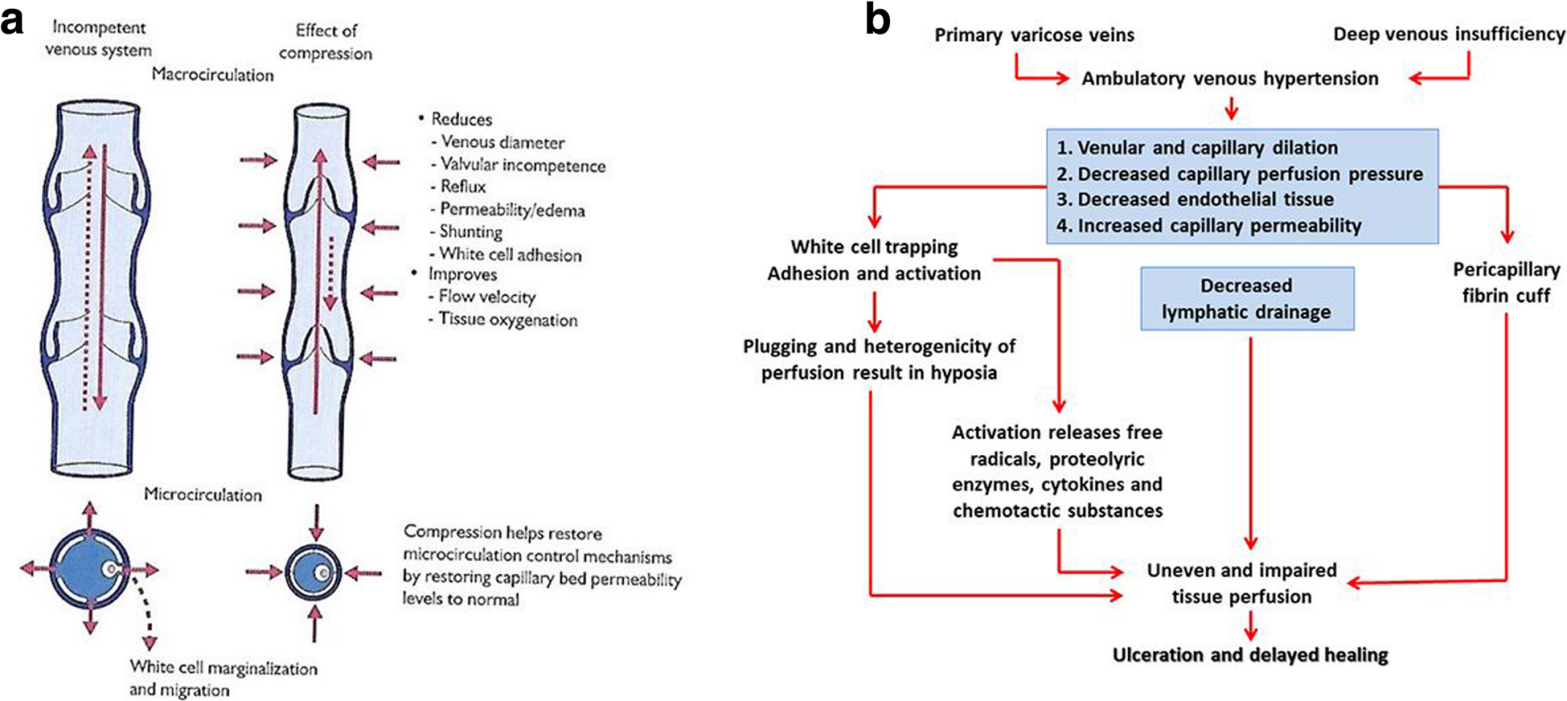
Cartoon of the pathophysiology of venous leg ulcers (VLU_s_). **a** The effects of valve incompetence and **b** the effects on tissues that lead to lipodermatosclerosis, cell death and ulceration. (Figures **a** and **b** were reprinted with permission from Mani R. Chronic Wound Management—the Evidence for Change, Parthenon Press 2002; copyright 2002 by Mani)

VLU_s_ are the result from the consequences of dysfunctional macro- and microcirculation [13, 14]. VLU_s_ are caused by unrelieved or ambulatory hypertension in the veins of the calf often resulting from deep venous thrombosis (DVT) that destroys venous valves rendering these incompetent and therefore unable to prevent venous backflow into the legs. High venous pressures are transmitted back to the capillaries and skin veins causing increased permeability, leakage and deposition of haemosiderin in the skin changing its texture and elasticity; legs become leathery to touch, brown and indurated. This condition is defined as lipodermatosclerosis, and it has been associated with leucocyte trapping and subsequent microcirculatory impairment, pericapillary cuffs that trap nutrients and other substances, and tissue hypoxia predisposing skin to cell death and ulceration [14] (Fig. 4). Congenital aplasia leading to venous valve dysfunction in turn resulting in venous hypertension and other sequelae described above can also result in venous ulcers. There is a lack of accord over what triggers venous ulcers. Patients frequently have a history of trauma, for example, scratching dry skin leaving a small hole or accidental skin damage resulting from banging a supermarket trolley into the legs. One patient complained her venous ulcer started from a scar after surgical removal of the long saphenous vein. Foot vein pressures in patients with venous disease significantly increase from normal pressures of 115 mmHg that is obtained in healthy individuals.

**Fig. 5:**
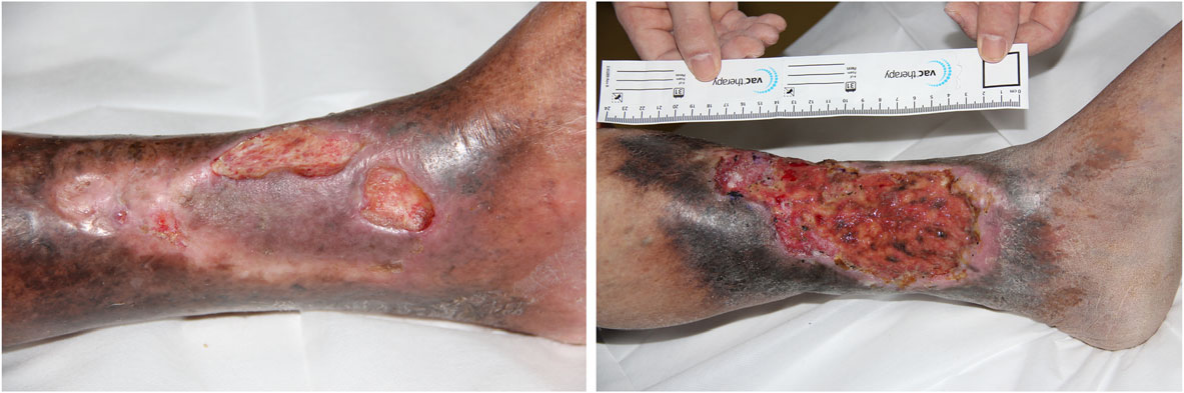
A mixed arterio-venous leg ulcers (VLU_s_) with dry, pigmented skin surrounding the ulcer (on the left) and an uncomplicated venous ulcer with a sloughy base (on the right)

Venous ulcers commonly carry a level of bioburden, though, occasionally, some VLU_s_ may get infected (Fig. 5). Biofilms are clinically suspected to be present on VLU_s_ though there are no reported data. These chronic wounds are often weepy, leaving the skin over the edges at risk of maceration.

### Diagnosis

Diagnosis of VLU_s_ is based on clinical examination followed by ultrasound Doppler measurement of ankle-brachial systolic pressure index (ABI or ABPI) [11, 15] to exclude arterial disease. Duplex ultrasound imaging measurements permit accurate measurement and location of sites of venous incompetence and are recommended where the equipment and trained sonographers are available [11]. Ultrasound measurement of ABI or ABPI is recommended in all guidelines since palpation of the pedal pulses dorsalis can and is difficult in a swollen foot. It is also known that some 5% of the population may have an unpalpable dorsalis pedis pulse.

Normal ranges of ABI or ABPI are as follows: 0.9–1.2 exclude arterial disease, ≤ 0.5 is consistent with the presence of severe peripheral ischaemia, ≥ 0.5 to ≤ 0.9 is consistent with the presence of peripheral arterial disease and ≥ 1.2 suggests a need to exclude aneurysmal changes or cardiovascular disease [11, 15].

### Management

Compression is the mainstay of management of VLU_s_ [11, 16] together with wound care. In earlier years, leg elevation during rest was recommended though this often proved difficult, and for these reasons, it is not included in current care practice. Compression may be delivered using multi-layered garments (4, 3 or 2) of which at least one must be elasticated capable of delivering external pressures of 35–40 mmHg at the ankles and decreasing to 17–20 mmHg at the level of the knee line [17]. Bandaging VLU_s_ is difficult and requires training and updated education to keep abreast of developments in these areas. Short stretch bandages (SSB) are effective and particularly helpful when patients are ambulant and able to pull their bandages up without help. In the UK, wrap-around bandages are usually removed and re-done after wound management by nurses. Such bandages are kept on when patients are in bed, so ensuring a degree of compliance. The downside of this approach, however, is the bulk of heavy bandaging limits the shoes that patients can wear. Compliance with bandaging is reported to be poor in the UK [12]. This raises the question as to how compression may be best delivered in warm countries in Asia, some parts of South America. This is a significant issue and raises the need for further research and development. Partsch et al. suggested that ‘static stiffness index’ (SSI) can be used to compare the efficacy of bandaging systems [18]. Partsch measured the pressure exerted by bandages on the skin and compared the changes between lying supine and standing when oedema will collect. This finding could be very important when designing bandaging systems more suitable to tropical climates. Mosti reported that Velcro-assisted device™ aided compression was more effective in removing oedema in the early phase of venous ulcer healing which could be very valuable in management than inelastic devices after a randomised controlled trial [19]. Elasticated stockings to deliver 30–40 mmHg are normally recommended for use as a maintenance measure after wounds are completely healed.

In cases when patients present with mixed arterio-venous ulcers, i.e. have both venous insufficiency and peripheral arterial disease, bandaging must be done with caution because excessive bandage pressure will make this type of wound worse. In general, when the ABI is in the range of 0.5–0.9, bandaging may be applied, but must be modified with lesser pressures. However, when ABI is < 0.5, bandaging is contraindicated, and treatment of wounds in this condition requires revascularisation to be first considered.

### Wound management

Wounds may be cleaned using sterile, warm-to-touch water and dressed preferably using a contact dressing that is easy to apply and pain-free to remove [11]. Most hospitals develop local protocols for wound care. Dressing change is determined by the need to keep the wound bed moist but free of exudate and the patient’s desire for cleanliness. Sometimes, patients find dressing changes painful; in practice, the use of eutectic mixture of local anaesthetics (EMLA^®^) cream around the ulcer is known to be helpful [11]_._ Where EMLA^®^ is not available, local suitable topical agents may be helpful. Specialised dressings (for example honey or silver) exist to cater for specific needs and should be used as defined [11].

Wound debridement is essential, based on a clinical decision, and is done by a surgeon, though in the UK, tissue viability nurse specialists may debride using sharps. The remarkable efficacy of technology for wound debridement using Negative Wound Pressure Therapy or Topical Negative Pressure is evidence-based [11].

### Wound outcomes

The outcomes of wound healing must be measured: this may be from contour surface area, from linear measurements across wounds or from wound perimeters [20]. Such measurements must be accurate and reliable and preferably done using non-contact methods to avoid cross infection. There is robust evidence to suggest that feedback of outcomes is beneficial to care of both VLU_s_ and diabetic neuropathic wounds [21]. Wound photography using simple cameras and planimetry (to measure area within) or dedicated wound cameras equipped with software is essential and extremely valuable. Such dedicated wound cameras produce high-quality images that are easily stored for later comparison though the cameras may be expensive. Mobile phones permit high-quality images to be transmitted from day care centres/patient homes/outpatient departments to centrally located wound departments. VLU_s_ healing is variable and unpredictable despite the use of standard care [11, 12].

### Does the use of adjuvants help standardised care?

Wounds being treated with standardised care are frequently slow to heal completely. When the delay is considered unduly long, or complications are suspected, it is advisable to revisit the diagnosis and check for underlying complications including the occasional presence of carcinoma. Once clinical confidence is restored, determine the value of adjuvants to promote healing. A plethora of adjuvants based on different transduction systems such as physical techniques, electromagnetic techniques and chemical and/or biological techniques have been reported [11]. Using these techniques, the evidence to improve or hasten healing compared with standardised care is variable but limited.

Therapeutic ultrasonic probes of low frequency and different intensities of both contact and non-contact (with skin) types have been rigorously tested in randomised controlled studies and have found significant benefits in improving healing rates of hard-to-heal VLU_s_. These findings were effective and permit this technique to be recommended [22]. Therapeutic ultrasound probes rely on sending bubbles of energy (pressure) which implode due to cavitation on surfaces.

Greer et al. [23] performed a systematic review of biologic dressings used to treat VLU_s_, diabetic foot ulcers (DFU) and ischaemic ulcers (standard care versus standard care plus biologics or, in some cases, biologic dressings versus advanced wound care). Primary outcome was complete healing, and time to complete healing was also examined as well as heterogeneity among studies. Evidence of healing was classified as high, moderate or low. Keratinocyte therapy was reported to offer moderate benefit to treat VLU_s_.

The clinical application of stem cells stirs controversies based on ethical concerns, age-related effects, decreased cell counts or the difficulties of fresh transplantation. However, with time, more cell lineages of interest appear. Mesenchymal stem cells (MSC), a kind of progenitor cell which is easy to isolate and expand, could enhance epithelialization and granulation tissue formation and neovascularization and synthesise essential growth factors and cytokines thus to improve wound healing. MSC has shown its potential as an effective therapeutic agent in various studies *in vivo* and* in vitro*. In a retrospective, non-randomised single-centre study of 67 chronic lower extremity wounds that included VLUs and DFU, human cellular repair matrix (h-CRM) was used with standardised care [24]. Treatment was standard care at weekly visits, regular debridement, offloading for DFU, compression for VLUs and h-CRM for wounds in > 4 weeks duration. After 12 weeks of study, 23/34 (67.6%) VLUs and 23/27 (85.2%) DFUs healed and no adverse events were noted. The results are interesting even though the lack of blinding and non-randomised selection render it impossible to exclude criticisms of bias: it certainly begs the question of follow-up studies which should also address recurrence.

Therapies of this kind are very expensive, future studies should investigate time to complete healing as well as recurrence within the context of standardised care.

### Does surgery help the healing of VLU_s_?

There is high-level evidence to report that surgery to treat superficial venous incompetence plus compression is recommended to prevent recurrence of VLU_s_ [11, 16]. This refers to ablative surgery which included venous stripping, ultrasound-guided foam sclerotherapy, endovenous laser ablation, radiofrequency ablation, mechano-chemical ablation and endovenous glue ablation. This may be particularly useful in cases where venous incompetence is limited to a superficial vein, for example the long saphenous vein. Even though such cases are the minority, it is essential to carefully diagnose and manage them. Duplex ultrasound measurement of venous reflux is a reliable test. Calf vein plethysmography, done using a tourniquet, can discriminate between the presence of deep and superficial vein incompetence or superficial vein incompetence alone. In other words, this test can identify whether the increased venous pressures in the calf are the result of deep and superficial venous incompetence or superficial venous incompetence alone. In general, when the latter is demonstrated, venous stripping (conventional open surgery) to close the incompetent vein (long and/or short saphenous vein), following complete wound healing, may be considered. Compression bandaging is used after surgery as reported by Barwell et al. [25]. However, nowadays, minimally invasive venous ablation methods, e.g. endovenous laser ablation, have replaced venous stripping in many areas. Recently, there was a trend toward venous ablation, especially endovenous ablation, together with compression bandaging to be performed while the patient had a frank or open wound to enhance wound healing [16]. Also, in cases where patients have pathological perforator incompetence, the perforator interruption is recommended to be done at the same time as superficial venous ablation is carried out [26]. Although this trend of thought needs to be tested against the background of new, robust evidence, this concept is relevant to venous ulcer management in Asia, where the weather is, almost always, warm and wet, forbidding the use of elasticated compression for long periods. The use of compression bandaging alone in Asia may not elicit compliance with patients.

### Does nutritional supplementation benefit VLU_s_?

Guest et al. [10] reported that nutritional deficiency odds ratio (OR) 0.53 (*p* < 0.001) and diabetes OR 0.65 (*p* < 0.001) were among the top independent risk factors for wound healing, others being dermatological and gastrointestinal symptoms. In a recent report, following a systematic review of the wound literature and meta-analysis of data, Ye and Mani reported that systemic and topical nutritional supplementation significantly benefitted patients with VLU_s_. Ye et al. analysed data from *N* = 23 randomised controlled studies and reported that overall, VLU_s_ patients significantly benefitted from nutritional supplementation [relative ratios (RR) = 1.44, 95% confidence intervals (CI) (1.31–1.59)] [27]. This report further stated that the systemic route was marginally superior to the topical one [systemic RR = 1.51, 95% CI (1.31–1.67), oral RR = 1.14, 95% CI (0.96–1.36)]. This analysis did not permit definition of which patient is to be selected for nutritional supplementation though routine nutritional assessment of patients with VLU_s_ may be a logical first step to be followed by designed trials with adequate sample size to test the efficacy of specific nutritional supplementation.

## Conclusion

The purpose of this paper was to review the pathogenesis and evidence for treatment of VLU_s_. VLU_s_ are the result of macro- and microvascular dysfunction, i.e. structural changes in the veins and tissues as well as haemodynamic changes which in skin breakdown over the ankles. Standardised care and mainstay of treatment for VLU_s_ have been defined based on evidence. It is clear from the evidence that our management of VLU_s_ must be improved and that suitable compression techniques for use in warmer countries as found in Asia and Africa need to be developed. There is also an unmet need for adjunct devices to speed wound healing.

## Abbreviations

CI: Confidence interval
DFU: Diabetic foot ulcers
OR: Odds ratio
RR: Relative ratio
VLU_s_: Venous leg ulcers
